# Identifying the effective concentration for spatial repellency of the dengue vector *Aedes aegypti*

**DOI:** 10.1186/1756-3305-5-300

**Published:** 2012-12-28

**Authors:** Nicole Achee, Penny Masuoka, Philip Smith, Nicholas Martin, Theeraphap Chareonviryiphap, Suppaluck Polsomboon, Joko Hendarto, John Grieco

**Affiliations:** 1 Department of Preventive Medicine and Biometrics, Uniformed Services University of the Health Sciences, 4301 Jones Bridge Rd, Bethesda, MD, USA; 2 Department of Labor-Occupational Health and Safety Administration, Health Response Team, 8660 South Sandy Pkwy, Sandy, UT, USA; 3 Department, Infectious Diseases Directorate, Viral and Rickettsial Diseases, U. S. Naval Medical Research Center, 503 Robert Grant Ave, Silver Spring, MD, USA; 4 Department of Entomology, Faculty of Agriculture, Katsetsart University, Bangkok, 10900, Thailand; 5 Department of Public Health and Preventive Medicine, Faculty of Medicine, Hasanuddin University, Makassar, 90245, Indonesia

**Keywords:** Spatial repellency, Air sampling, *Aedes aegypti*, Mosquito behavior, Experimental hut, Chemical concentration, DDT, Metofluthrin

## Abstract

**Background:**

Current efforts are underway to quantify the chemical concentration in a treated air space that elicits a spatial repellent (deterrent) response in a vector population. Such information will facilitate identifying the optimum active ingredient (AI) dosage and intervention coverage important for the development of spatial repellent tools – one of several novel strategies being evaluated for vector-borne disease control. This study reports initial findings from air sampling experiments conducted under field conditions to describe the relationship between air concentrations of repellent AIs and deterrent behavior in the dengue vector, *Aedes aegypti*.

**Methods:**

Air samples were taken inside and outdoors of experimental huts located in Pu Tuey Village, Kanchanaburi Province, Thailand in conjunction with mosquito behavioral evaluations. A mark-release-recapture study design using interception traps was used to measure deterrency of *Ae. aegypti* against 0.00625% metofluthrin coils and DDT-treated fabric (2g/m^2^) within separate experimental trials. Sentinel mosquito cohorts were positioned adjacent to air sampling locations to monitor knock down responses to AI within the treated air space. Air samples were analyzed using two techniques: the U.S. Environmental Protection Agency (USEPA) Compendium Method TO-10A and thermal desorption (TD).

**Results:**

Both the USEPA TO-10A and TD air sampling methods were able to detect and quantify volatized AIs under field conditions. Air samples indicated concentrations of both repellent chemicals below thresholds required for toxic responses (mortality) in mosquitoes. These concentrations elicited up to a 58% and 70% reduction in *Ae. aegypti* entry (i.e., deterrency) into treated experimental huts using metofluthrin coils and DDT-treated fabric, respectively. Minimal knock down was observed in sentinel mosquito cohorts positioned adjacent to air sampling locations during both chemical evaluations.

**Conclusions:**

This study is the first to describe two air sampling methodologies that are appropriate for detecting and quantifying repellent chemicals within a treated air space during mosquito behavior evaluations. Results demonstrate that the quantity of AI detected by the mosquito vector, *Ae. aegypti*, that elicits repellency is far lower than that needed for toxicity. These findings have important implications for evaluation and optimization of new vector control tools that function through mosquito behavior modification as opposed to mortality.

## Background

With the charge of malaria elimination and eradication before us, and the scale of scope of dengue continuing to grow, new tools and/or further optimization of current products will be required to meet public health demands. An emphasis must be placed on optimizing current vector control tools and outlining strategic plans for the development of effective novel products [1]. While the use of long-lasting insecticide treated bed nets (LLINs), indoor residual sprays (IRS) and space spraying have demonstrated substantive disease impact in organized campaigns, these interventions face challenges of user compliance and/or threats of reduced efficacy due to insecticide resistance. In addition, a thorough evaluation of the role of supplementary (or stand-alone) tools targeting those vectors not affected by either LLINs or IRS is needed. This is particularly true for outdoor disease transmission settings as well as for day biting vectors, perhaps most evident in the case of dengue.

One such group of tools of interest is spatial repellent products that function in the volatile phase to reduce human-vector contact by causing movement away from a chemical stimulus, interfering with host detection (attraction-inhibition) and/or feeding response. One measurable outcome of spatial repellency is deterrency, or the ability to prevent vectors from entering a treated space (inside a home or outdoor area) [2]. Ideally, the product would operate through a passive delivery system (not require heating to activate chemical dispersal) and be designed in formats that are not only effective but desirable and affordable to the end user. The result would be a highly sustainable product that adds an additional layer of bite protection beyond standard IRS and ITN platforms and provides a protective barrier in outdoor transmission sites that have previously been unattainable. The target product profile also includes release of AI for several months to increase duration of bite protection. The key characteristic that separates spatial repellents from current chemical adult control tools – LLINs and IRS – are that they provide protection at concentrations much lower than recommended field application rates [3]. This is because the outcome measure is not toxicity but rather behavior modification. Direct benefits of such a tool would be: 1) the delayed onset for selection of insecticide resistance (minimal selection pressure due to reduced toxicity) thereby extending the products effective duration, 2) reduced mammalian toxicity due to application at low chemical concentrations, 3) delivery formats that eliminate the need for direct vector contact to be effective thus allowing for point source application which can facilitate product distribution.

However, the critical path of development of spatial repellent tools includes correlating the effective concentration of AI in a treated air space with deterrent mosquito behaviors. Methodologies for conducting these evaluations will allow identification of minimum effective concentrations required for creating vector free spaces. Additional parameters such as the length of time that an effective concentration remains in the air space and the distance at which the effective concentration can be detected will facilitate the optimization of the delivery format for such a tool. The objective of the current study was to determine appropriate methodologies for detecting and quantifying the air concentration of two repellent chemicals, metofluthrin and DDT, under field conditions and describe the relationship between AI concentrations with a reduction in *Ae. aegypti* (recently also classified as *Stegomyia aegypti*[4]) entry into the treated space.

## Methods

**Figure 1:**
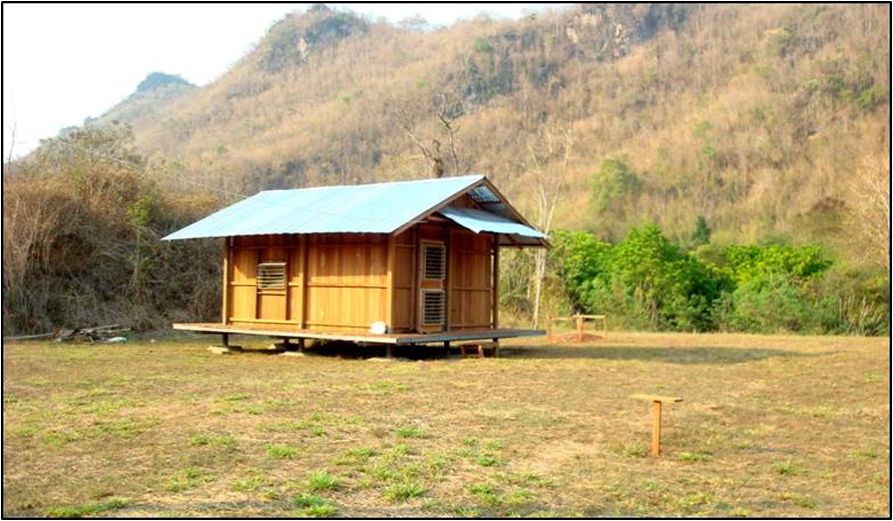
**Experimental huts.** Evaluations were conducted using experimental huts in Pu Teuy Village Thailand.

The study was conducted using experimental huts (50m^3^) in Pu Teuy Village, Sai Yok District in Kanchanaburi Province, Thailand (14020’11”N, 98059’45”E). Located approximately 150km Northwest of Bangkok, Pu Tuey is inhabited by approximately 1500 individuals in 110 houses and is surrounded by small agricultural plots. The experimental huts are positioned in the center of the village, approximately 45m from each other and 800m from the nearest home (Figure 1). Baseline evaluations were performed prior to treatment to ensure huts were equal in attractiveness (data not shown). Study protocols were reviewed by Kasetsart University and the Uniformed Services University of the Health Sciences (USUHS) scientific review committees with ethical approvals provided by corresponding Institutional Review Boards accordingly.

### Test compounds and study design

Technical grade DDT −1,1 Bis (4-chlorophenyl)-2,2,2- trichloroethane, (CAS 50-29-3,Sigma-Aldrich) and metofluthrin coils (0.00625%; SC Johnson & Co.) were evaluated in separate experiments. These chemicals were selected based on current World Health Organization Pesticide Evaluation Scheme (WHOPES) recommendations for vector control [5] and/or use in currently available mosquito control products marketed as repellents (i.e., mosquito coils). A total of four and three replicates (days) were performed for DDT and metofluthrin evaluations, respectively.

**Figure 2:**
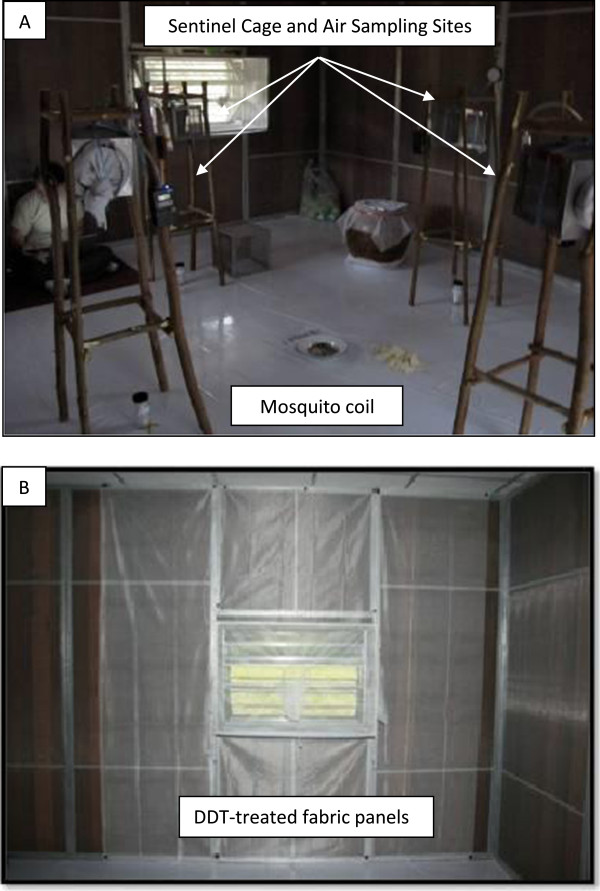
**Chemical treatment.** (**A**) Coils dosed with 0.00625% metofluthrin (active coil) or inert ingredients alone (blank coil) were positioned at the center of treatment and positive control huts, respectively. (**B**) During DDT evaluations, polyester panels treated at 2g/m^2^ were affixed to interior hut walls.

Evaluation of metofluthrin was conducted in January 2010 using three experimental huts designated as: negative control (no coil), positive control (blank coil) and treatment (active coil). Active coils dosed with 0.00625% metofluthrin and blank coils, containing inert ingredients alone, were provided by S.C. Johnson & Son, Inc. to the Thailand study site. During each experimental day, a single active (treatment) or blank (control) coil was placed within a 2 cm X 30 cm (Depth X Width) enamel bowl positioned on the floor at the center of the experimental huts (Figure 2). Coils were lit at 0530 h by collectors within the hut and were replaced at 1200h with a new coil to standardize burn time (0530–1200h and 1200-1800h), control for potential coil failure, as the coils were labeled for 6 hour use, and ensure an uninterrupted release of AI. The coil was allowed to burn for 30min prior to initiating air sampling and evaluation of mosquito behavior. For all test days (n=3), the treatment coil was evaluated side-by-side with matched positive and negative control huts.

Evaluation of DDT was conducted in October 2010 using three experimental huts designated as: negative control (containing solvent-treated fabric), and two treatments (containing chemical-treated fabric). Polyester netting panels (mesh size 24 × 20 per inch, Bioquip Inc., Rancho Dominguez, CA) representing 50% of the total interior wall surface area of the experimental huts were treated with analytical grade DDT (CAS 50-29-3; 1,1 Bis(4-chlorophenyl)-2,2,2-trichloroethane, 98%)(Sigma-Aldrich) solution using previously established protocols [3] at 2 g/m^2^ field application rate [5]. Netting panels for the control hut were treated with acetone diluent only. Treatment and control panels were treated under laboratory conditions and allowed to dry under a chemical fume hood. When dry, the panels were immediately wrapped in aluminum foil, placed in sealed plastic bags and transported on ice to the field site. Netting panels were positioned inside the experimental huts 12 h prior to the first day of testing by attaching to metal frames aligned on interior walls using magnets (Figure 2). Panels were placed around the perimeter of the windows and doors as these represented key portals for mosquito entry. For all test days (n=4), the treatments were evaluated side-by-side with the matched negative control hut.

### Mosquito behavior evaluation

The deterrent, knock down (KD), and 24h mortality responses of *Ae. aegypti* test populations upon exposure to 0.00625% metofluthrin coils and DDT-treated netting were quantified using a mark-release-recapture study design. Evaluations were performed following previously described protocols [3]. Briefly, three experimental huts, constructed using materials that matched indigenous Thai homes, were affixed with window and door interception traps that functioned to capture entering mosquitoes originating from outside the hut [6]. Each hut is 4 m × 5 m × 2.5 m, with a total internal volume of 50 m^3^. Test cohorts of 100 female, 4–7 day old, 24h sugar-starved *Ae. aegypti* mosquitoes were uniquely marked with fluorescent powder (BioQuip Products, Inc., Gardena CA), color-coded by hut and simultaneously released at 0530h from fixed positions 10m outside of each hut. Two collectors were positioned inside each hut to serve as collectors and to generate host cues. Collector teams were rotated among huts each day at 1200h. Interception traps were sampled for entering mosquitoes using manual aspiration every 20min until 1800h.

The effect of chemical in the air to cause mosquito KD (the inability of a mosquito to right itself or fly) was observed every 20 min using sentinel cohorts. Groups of 10 female *Ae. aegypti*, originating from the same colony as the release test population, were placed into 10 cm^3^ mesh cages positioned ~1 m from the floor both inside (metofluthrin and DDT evluations) and outside (metofluthrin evaluations only) portals of entry of treatment huts. The location of KD sentinel cohorts matched locations of air sampling (Figure 2). For metofluthrin evaluations, only one KD sentinel cohort was placed directly on the floor in the center of the negative control hut (i.e., no coil). All sentinel mosquito cohorts were replaced with fresh mosquito populations at 1200h each day of evaluation with both sentinel cohorts and mosquitoes collected from interception traps held overnight in the field insectary to monitor 24h mortality. Wind velocity (mph) was recorded every 20 min outside windows and doors of the treatment hut during metofluthrin evaluations using a hand-held weather tracker device (Kestrel 4000, KestrelMeters, Birmingham, MI). Wind velocity measurements were aggregated into two-6h sampling periods (0600-1200h and 1200-1800h) then averaged for each of the first time period designated as ‘AM’ and the second time period as ‘PM’.

### Air sample collections and processing

#### Metofluthrin

**Figure 3:**
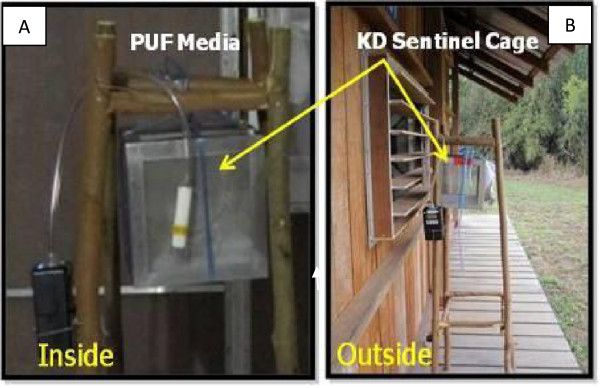
**Knock down observation.***Aedes aegypti *knock down responses were observed in sentinel mosquito cohorts positioned (**A**) inside (metofluthrin and DDT evaluations) and (**B**) outside (metofluthrin evaluation only) portals of hut entry at matched air sampling locations.

Air samples were collected at four indoor and four outdoor positions at the treatment hut corresponding to each KD sentinel cohort site (3 windows and 1 door) (Figure 3). Samples were taken in 6h periods from 0600h-1800h using sorbent tubes (20 mm ID × 10 cm glass) packed with purified polyurethane foam media (PUF) (22-mm ID × 7.6 cm cylinder). A 5L/min < ± 5% volume air was pumped through the media using continuous-flow sampling pumps (AirCheck XR5000). The sorbent tubes and pumps were calibrated (in-line) prior to sample collections. A dry volumetric calibrator (Defender 510, Bios International, Butler, NJ), capable of 5L/min was used to verify airflow through the sorbent tube. Due to limitations in the number of PUF vials available (48), sampling periods were divided into two-6 h sampling periods (0600-1200h and 1200-1800h) with the first time period desingated as ‘AM’ and the second time period ‘PM’. Sampling was synchronized among all air sampling stations to start 30 min after coil burning (i.e., 0600h). At the end of each sampling period, PUF vials were replaced with new vials and immediately transferred to 4°C conditions until processing. Samples were labeled according to pump identification number, portal position (window number and/or door), time period of sampling, total pump running time and indoor or outdoor location. Processing of PUF media was conducted at the ALS Laboratory in Kuala Lumpur Indonesia following United States Environmental Protection Agency (USEPA) Compendium Method TO-10A (American Society for Testing and Materials (ASTM) Method D4861-94) standard protocol [7]. This particular processing method was selected based on preliminary analyses of metofluthrin quantification from SC Johnson Co. laboratories (Ken Welch, pers comm). Levels of metofluthrin air concentration were reported for each replicate day according to sentinel site (i.e., window 1–3 and door) and indoor/outdoor location then aggregated by AM or PM.

### DDT

Air samples were collected from the center of the control hut (Hut A). Air samples from treatment huts (Hut B and C) were collected from five indoor positions corresponding to each KD sentinel cohort site at the windows (3), door (1) and a center location (1). Samples at all huts were taken hourly from 0600h-1800h using metal tubes (89 mm X 4 mm i.d. X 6.4 mm o.d.) packed with 200 mg of Tenax-TA adsorbent (Markes International, Ilantrisant, UK). Prior to sample collection, tubes were conditioned at 300°C for 20 min with a constant N_2_ stream (30 mL/min). Air samples were collected with personal air sampling pumps (Model 222, SKC inc., Eighty Four, PA) calibrated before and after sample collection using a device to measure volumetric flow rate (Defender 510, Bios International, Butler, NJ). In the field, pumps were set to operate with flow of 200 mL/min (± 2 mL/min) collected during 60 min sampling intervals (i.e., sample total ~12L) then analyzed onsite within 96 hours of collection using a previously described thermal desorption (TD) method [8]. Briefly, sample tubes were thermally desorbed using a Unity 2 thermal desorber (Markes International, Ilantrisant, UK) and analyzed by gas chromatography–mass spectrometry with a field portable instrument (Model 5975T, Agilent Technologies, Santa Clara, CA). Standard samples prepared at Katsetsart University were used to calibrate the instrument in the field.

### Data analysis

The total number of *Ae. aegypti* collected from interception traps affixed to experimental huts was used to calculate percent reduction in *Ae. aegypti* entry (i.e., deterrency) as compared to control using (100 x (E_c_-E_t_)/E_c_); where E_c_ and E_t_ represent total mosquitoes entering the control and treatment huts, respectively. Percent deterrency was compared among huts using chi-square analyses with α set at 0.05 (GraphPad Software, Inc., La Jolla, CA). Indoor and outdoor metofluthrin air concentrations, as well as AM and PM samples, were compared using the Mann–Whitney Rank Sum Test. The mean concentration of airborne DDT measured in the two treatment huts was also compared using the Mann–Whitney Rank Sum Test in Sigma Plot for Windows (Version 11.0, Systat Software, Chicago, IL). A p value of less than 0.05 was considered to indicate statistical significance.

## Results

### Mosquito behavior

Overall, 0.00625% metofluthrin coils elicited a 58% (20/48) decrease in *Ae. aegypti* entry in the treatment hut compared to the negative control hut (i.e., no coil) which had a total of 48 marked mosquitoes entering (Table 1). The positive control hut (i.e., with blank coil) showed a total 48% (25/48) deterrent effect compared to the negative control. Deterrency in response to blank and 0.00625% metofluthrin coils was signifcant compared to control (P=0.008; df=2; *χ*^*2*^ = 14.387). Percent KD observed in *Ae. aegypti* sentinel cohorts inside the hut containing 0.00625% metofluthrin coil was higher (1.7%; 4/240) than both the positive (0.8%; 2/240) and negative controls (0%; 0/60); however, minimal KD occurred overall during the evaluation with a total of only 6 and 5 observations from indoor and outdoor cohort positions, respectively (Table 1). Percent KD at each coil-containing hut was similar between indoor and outdoor sentinel positions. Mortality rates of sentinel cohorts positioned inside the hut containing blank coils (5%; 13/240) and 0.00625% metofluthrin coils (5%; 12/240) were also similar (data not shown). Percent of 24h mortality from outdoor cohorts was slightly higher (9%; 23/240) but again similar for both coil-containing huts. A total of 8% (5/60) mortality was observed from sentinel cohorts inside the control hut (data not shown). Outdoor wind velocity recorded during metofluthrin evaluations was minimal, ranging from 0.0135-1.55 mph. The mean of 0.85 mph recorded during the PM sampling period was slightly greater as compared to the mean 0.29 mph for AM (data not shown). Although total mosquitoes collected during the three day evaluation at the treament hut were too low for rigorous analyses, in general, highest interception trap collections were made at portals of entry with lowest metofluthrin air concentration (Additional file 1: Figure S1).

**Table 1:** **Total marked *Ae. aegypti***^**1 **^**recaptured, active ingredient (AI) air concentration, knock down and deterrency during field evaluations**

	**Mosq. recaptured**		**AI air concentration (μg/um**^**3**^**)**^**3**^			**% Knock down**^**4**^		**Deterrency**			
**Treatment**	**No. Mosq.**	**%(n/Total release)**^**2**^	**Range**		**% Quantifiable samples (n/Total)**	**In (n/Total)**	**Out (n/Total)**	**Total entry**	**% (n/Total control)**	**P**^**5**^	
			**(Mean±SD)**								
			**In**	**Out**							
No coil	48	5.3	-	-	-	0	-	48	0	
(Hut A)						(0/60)				
Blank coil (Hut B)	25	2.8	-	-	-	0.8	0.8	25	48	0.008
						(2/240)	(2/240)			
										(*x*^2^=14.387)
0.00625%	20	2.2	0.001-0.11	0.001-0.03	100(48/48)	1.7	1.3	20	58	
Metofluthrin										
			(0.058±0.03)	(0.019±0.01)						
						(4/240)	(3/240)			
(Hut C)										

Chemical-free netting (Hut A)	100	8.3	ND-1.22	-	10(1/10)*	0	-	100	0	
			(n/a)			(0/320)				
2g/m^2^ DDT^6^ (Hut B)	30	2.5	ND-1.57	-	65(11/17)	0	-	30	70	<0.0001(*x*^2^=45.186)
			(0.74±0.45)†			(0/320)				
2g/m^2^ DDT	47	3.9	ND-3.98	-	93(28/30)	0	-	47	53	
						(0/320)				
(Hut C)			(1.42±0.96)							

^1^4-7 day old, 24h sugar-starved females.

^2^Metofluthrin evaluation = 900; DDT evaluation = 1200.

^3^Both indoor/outdoor air sampling during metofluthrin evaluations; only indoor air sampling during DDT evaluations; ND = not detectable.

^4^Observations from sentinel cohorts within screened cages positioned at air sampling locations.

^5^Chi-square analyses with α = 0.05 and 2 degrees of freedom.

^6^Treated-netting panels comprising 50% surface area of interior hut walls.

^*^One sample; a labeling error is suspected.

^†^Median concentration of airborne DDT significantly different between treatment huts (p<0.05) based on Mann–Whitney Rank Sum Test.

For DDT evaluations, there was a 70% (30/100) and 53% (47/100) reduction in *Ae. aegypti* entry at the two treated huts as compared to the matched control (Table 1). Deterrency in response to DDT-treatment was signifcant compared to control (P<0.0001; df=2; *χ*^*2*^ = 45.186). No KD or 24h mortality responses were observed from sentinel cohorts fixed at the indoor air sampling positions. Mortality rates in *Ae. aegypti* populations captured from interception traps affixed to huts containing DDT-treated netting was minimal (0.9% overall) and similar to that observed in the control hut (0%; data not shown).

### Chemical analyses

A total of 48 air samples were analyzed from inside and outdoors of the experimental hut containing 0.00625% metofluthrin coil (Table 1). metofluthrin coil over the three day evaluation, air concentrations at the treatment hut ranged from 0.001 – 0.11 μg/m^3^ inside and 0.001 – 0.03 μg/m^3^ outdoors. The mean indoor metofluthrin air concentration (0.058 μg/m^3^) was significantly higher than that detected outside the treatment hut (0.019 μg/m^3^) (P<0.001). Mean indoor concentrations were significantly higher in the AM (0.081 μg/m^3^) as compared to the PM (0.036 μg/m^3^) (P=0.001); however, metofluthrin concentrations from air samples collected outside the treatment hut were similar between AM and PM sampling periods (0.019 μg/m^3^) (P=0.9761; data not shown). In general, the greater the wind velocity recorded at a given outdoor KD sentinel cohort site, the lower the metofluthrin concentration detected from air sampling; however, records of wind velocity were low overall during evaluation (Additional file 1: Figure S1).

During DDT evaluations, a total of 57 air samples were analyzed from inside experimental huts (Table 1). Airborne DDT concentrations ranged from non-detectable (ND) in the control hut to 3.98 μg/m^3^ in treatment Hut C with means of 0.74 μg/m^3^ and 1.42 μg/m^3^ for Hut B and Hut C, respectively (Table 1). The median concentration of airborne DDT in Hut B (0.66 μg/m^3^) differed significantly from that of Hut C (1.06 μg/m^3^) (P<0.05). Only 1 of the 10 samples processed from the control hut (i.e., chemical-free treatment) indicated presence of DDT; however, a labeling error is suspected. In addition to a gas chromatography peak for DDT, three other DDT-related gas chromatography peaks were noted during sample processing (data not shown). The earlier eluting peaks are likely DDT degradation products o, p′ DDD, p, p′ DDD, and the DDT isomer o,p′ DDT based on the elution order and corresponding full scan mass spectra.

## Discussion

One of the key issues in the development of novel chemical approaches for vector control – to include spatial repellency – is defining the underlying mechanism that results in the reduction of mosquito densities within a treated space and therefore prevention of human-vector contact. Both toxic (mortality) and sublethal (repellency) actions will produce a vector free space, however, one is due to a direct killing action while the other is not. If the use of spatial repellents for disease prevention is to be supported, and thereby drive screening paradigms for novel chemical modes of action [2], clear evidence must be presented that: 1) the concentration of a repellent AI in a treated air space is below toxic thresholds and 2) that sublethal air concentrations reduce human-vector contact to include measures of deterring vector entry into the treated space. In essence, correlating AI concentration with key entomological measures of disease impact (i.e., human-vector contact) will guide standardization for the evaluation of spatial repellent products [8]. This study outlines initial steps in determining methodologies and study designs for achieving this goal.

Our initial air sampling studies conducted in the laboratory against DDT indicated a thermal desorption (TD) method could detect volatilized DDT within the air space of a mosquito behavior monitoring assay [9]. Results from these experiments also showed that the TD method was able to detect a gradient of DDT air concentration among the three chambers (unpublished data). Based on the ability of the TD technique to detect and quantify DDT, as well as the relatively quick turn-around time for processing air samples, this technique was selected as one possible method for field validation in the current study. The second air sampling method employed here, the USEPA TO-10A, uses a chemical rather than heat desorption process to collect AIs from a set volume of air and has been validated for the atmospheric determination of pyrethrins and the pyrethroids d-allethrin, d-trans-allethrin, permethrin, resmethrin, bifenthrin, prallethrin, transfluthrin and metofluthrin - all of which are currently registered repellent actives [7]. Both air sampling and chemical analysis methods utilized in the current study may have utility in future spatial repellent evaluations. A direct comparison of the performance (LOD-limits of detection/LOQ-limits of quantification) of the two methods is not appropriate as different analytes were measured and there are a number of parameters that can be varied in the TO-10A method (detector type, final volume following solvent extraction, injection volume, and field vs lab analysis) that can impact the LOD and LOQ. Thermal desorption methods, similar to the one described here and elsewhere [10], demonstrate short term (≤8h), low-volume (<1m^3^) sampling can be used to measure the concentration of semi-volatile compounds allowing hour by hour measures of spatial repellent generation. The greatest limitation of these TD methods is they have not been assessed with the same rigors as the USEPA TO-10A method. Likewise, while the TO-10A method represents a standard air sampling and analysis method published by the USEPA, hourly variations in the concentration of semi-volatile SR cannot be reported as the method is intended for sampling periods of no less than 4h [7]. Such characteristics, in addition to cost and logistics, are important to consider when evaluating chemical presence under field conditions.

A 58% reduction in *Ae. aegypti* entry occurred in the hut containing a 0.00625% metofluthrin coil but deterrency, albeit attenuated, was also observed using a blank coil (48%). This indicates that smoke alone has some effect on mosquito entry. While similar effects have been reported through the use of traditional repellent methods (i.e., burning of organic material, etc.), additional replicates beyond that performed in the current study are required to fully determine any significant differences in mosquito behavior in response to blank and metofluthrin coils. The total number of marked *Ae. aegypti* collected at any of the huts was low (range of 20–48) during metofluthrin evaluation, therefore rigorous analyses to correlate mosquito entry with chemical concentration in the air space could not be performed. In addition, only one sentinel cohort was positioned in the hut without a coil versus four used in the huts containing active and blank coils therefore direct comparisons of KD and 24h mortality between treatment and negative control may be biased. However, observed knock down and 24h mortality were similar between the two coil-containing huts (i.e., active and blank) indicating no greater toxic effect in the chemical treated space as compared to that containing inert ingredients alone. In addition, overall KD inside the hut containing the metofluthrin coil was minimal although AI was detected in the indoor air space at higher concentrations than outdoors. Combined, these results suggest that toxicity is having little, if any, impact on the number of *Ae. aegypti* entering the hut containing a 0.00625% metofluthrin coil and indicates deterrency measured was due primarily to a sublethal repellent effect. This conclusion is supported by the fact that air samples collected adjacent to both indoor and outside KD sentinel cohort sites measured metofluthrin at means well below the toxic ‘effective’ rate of 1.0 μg/m^3^ as specified by the manufacturer (Ken Welch, pers comm).

DDT evaluations also indicated a significant deterrent effect in *Ae. aegypti* release populations in response to chemical treatment as compared to a chemical-free control. This response occurred in the absence of KD observations in sentinel cohorts. Given the THAI *Ae. aegypti* strain used in the current study have been characterized as DDT resistant [11], the lack of KD and mortality was not unexpected but instead strengthens the conclusion that a sublethal repellent mechanism was responsible for the reduction in mosquito entry rather than toxicity. Results also showed a difference in the median concentration of airborne DDT measured between the two treatment huts despite the fact that both received the same treatment. The concentration of airborne DDT is temperature dependent and therefore can vary significantly over the range of temperatures that may occur during field evaluations [12]. The significant difference in DDT air concentration between treatment huts therefore, was most likely due to the comparison of samples that were collected at various times (i.e., different hours) and locations (center vs. window etc.) and therefore at potentially different specific ambient temperatures and relative humidity conditions. The influence of microclimate conditions and airborne repellent chemical should be explored further in subsequent field studies. Regardless, the detection of DDT at the two treatment huts indicated AI was within the air space and the quantification was higher than that observed in the control, chemical-free hut.

The authors note that the placement of metofluthrin and blank coils (i.e. center of the floor) in relation to air sampling and KD sentinel cage locations (i.e. 1m above the floor) may have biased chemical particle dispersion and/or air space dosing and thereby mosquito KD outcomes. We chose this coil positioning to determine the atmospheric concentration of metofluthrin delivered from a mosquito-coil product based on one probable consumer-use scenario in which coils would be positioned on the floor of a house. The KD sentinel cohorts – and therefore air sampling – were fixed at the mid-way height of the windows and door of the experimental huts to reflect potential flight patterns during initial mosquito entry into the hut. In addition, air sampling and KD measurements directly outside the doors and windows during metofluthrin evaluations could have been confounded by interception traps and/or cages used to hold sentinel mosquito populations. The mesh netting on these structures could have prevented passage of some chemical particles and thereby diluted the chemical concentration and/or KD effects. However, we chose to use sentinel cages to evaluate KD behavioral effects as it represents a standard methodology in chemical exposure tests as outlined in WHO efficacy guidelines [13]. Regardless, future studies could explore KD sentinel structures that better match chemical exposure under free-flying field conditions. Other methodologies to evaluate KD outside the huts could include white plastic sheeting at various distances away from the treated space although the challenge of such an approach will be to minimize the disturbance of incapacitated mosquitoes by predators.

Most important to the goal of the study, both DDT and metofluthrin were detectable and quantifiable inside treated experimental huts and resulted in reduced *Ae. aegypti* entry into corresponding treated spaces. In addition, both air sampling techniques employed in the current study were able to measure chemical actives in the air space, and indicated concentrations below thresholds required for toxic responses (mortality). In fact, spatial repellent (i.e., deterrent) responses using 0.00625% metofluthrin coils occurred at AI air concentrations representing ~2.8ppt (0.06μg/m^3^) and ~0.9ppt (0.02μg/m^3^) inside and outside, of huts, respectively, and at ~35ppt (0.74μg/m^3^) inside a hut treated with 2g/m^2^ DDT at 50% surface area coverage, highlighting the sensitivity of potential mosquito receptors that may drive behavioral modifications that underlie spatial repellent mechanisms of action [14]. Definitive correlations between spatial repellent versus toxic properties of these two test chemicals that result in a vector reduced area will require more evaluations similar to those reported here as the limited number of replicates performed in the current study precluded rigorous analyses.

## Conclusions

This study has demonstrated that the quantity of active ingredient (AI) detected by the mosquito vector, *Ae. aegypti*, that elicits repellency is far lower than that needed for toxicity. This association has not been rigorously measured under field conditions before. These findings have important implications for the evaluation of new tools for vector control, to include spatial repellents, because standard testing methods currently focus only on toxicity of AIs rather than behavioral effects that might also have a great effect on disease transmission by breaking human-vector contact. In addition, both air sampling methodologies utilized here were able to detect AI within corresponding treated spaces validating their application during mosquito behavior field evaluations. The next steps in advancing our understanding of spatial repellent mechanisms of action include simultaneous air sampling and entomological assessments at distances away from a treated space. Such studies will continue to address program-relevant issues such as the outdoor area protected by a given product at a given chemical concentration useful for guiding coverage thresholds required for a spatial repellent intervention.

## Competing interests

There are no competing interests. The contents are the responsibility of the authors and do not necessarily reflect the views of the United States Government or the Department of Defense (NA, JG).

## Authors’ contribution

NA drafted the manuscript and with JG designed the study with the aide of Ken Welch and Maude Meier of SC Johnson & Co. Inc. NA, JG and PM analyzed the data and generated tables and figures. SP and JH conducted air sampling and mosquito behavior evaluations. NM and PS coordinated the DDT evaluation. All authors read and approved the final version of the manuscript.

## Supplementary Material

Additional file 1 **Figure S1.** Summary graphic of *Ae. aegypti* entry (counts), wind (mph) and metofluthrin air concentration (μg/m^3^) aggregated by AM (0600h-1200h) or PM (1200h-1800h) time periods for each day of evaluation. Click here for file
