# Supplementary figures and images for: Identifying the effective concentration for spatial repellency of the dengue vector Aedes aegypti
Source: Parasit Vectors. 2012 Dec 28;5:300. doi: 10.1186/1756-3305-5-300 (PMC3543351; doi:10.1186/1756-3305-5-300)

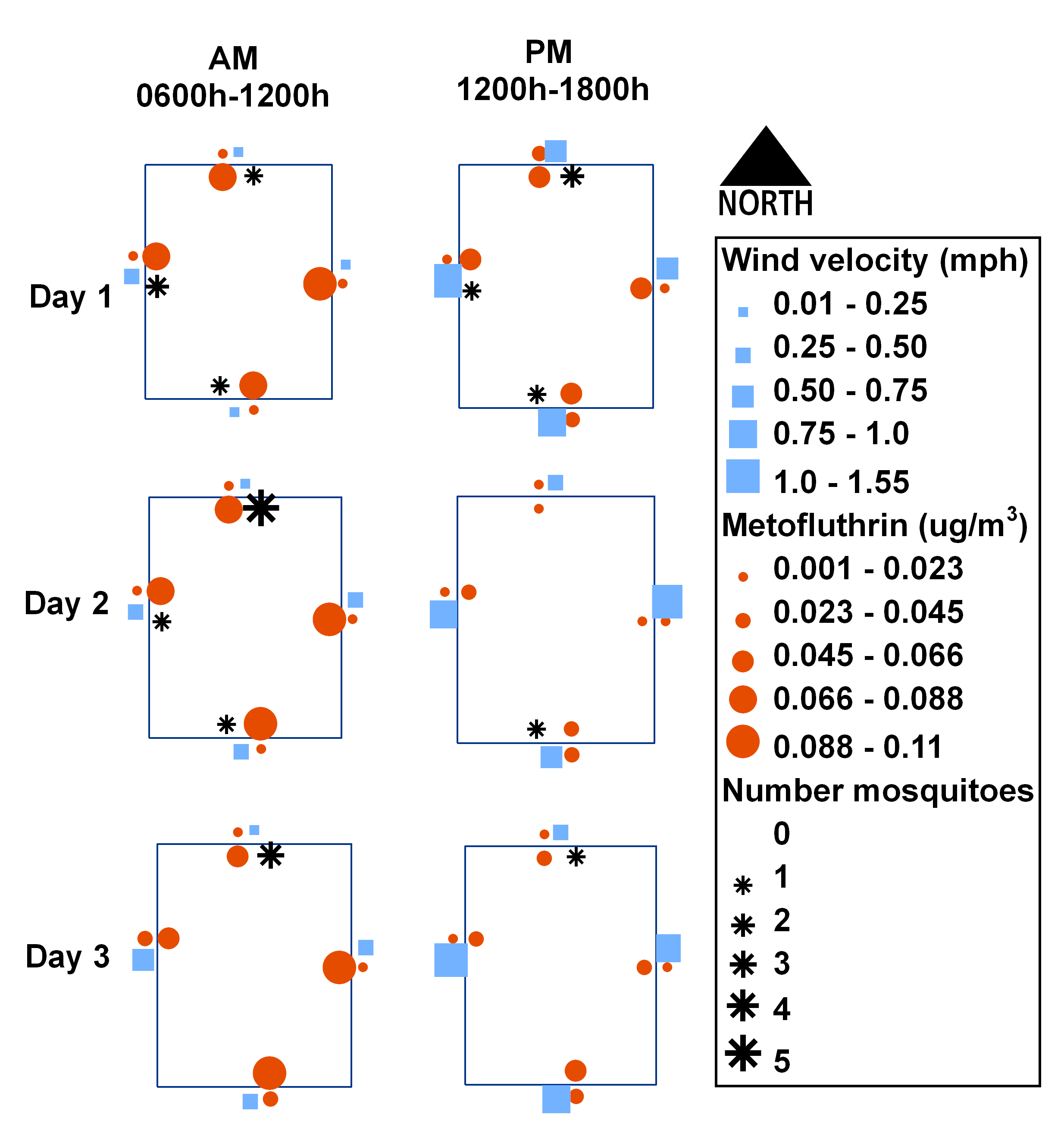

Supplement: Additional file 1 — Figure S1. Summary graphic of Ae. aegypti entry (counts), wind (mph) and metofluthrin air concentration (μg/m3) aggregated by AM (0600h-1200h) or PM (1200h-1800h) time periods for each day of evaluation. [file 1756-3305-5-300-S1.tiff]
